# Convention of Delegates from Western Schools

**Published:** 1849-03

**Authors:** 


*Convention of Delegates from Western Schools.*—We are authorised by the Faculty of the school with which we are connected, to state, that they will cheerfully unite in the proposed convention of western schools, provided *all* the schools interested will do so. If it be generally preferred that the convention should be held on the last Tuesday of April next, as suggested by the editor of the Western Lancet, the Buffalo School will not oppose it, but the plan of holding the meeting at Boston before, after, or during the session of the American Medical Association, would be more acceptable. The objection that a meeting of western delegates should be held on western soil, does not strike us as possessing much force; on the other hand, it seems to us there would be an advantage in comparing views with those connected with eastern schools, and it is not improbable that a convention of the schools of all sections might be held at the same time and place, the advantages of consultation and united effort being thereby extended. The last Tuesday of April is also objectionable because it will interfere with attendance at the National Association. An earlier or later date would have been preferable. We hope the suggestion by us that the meeting be held at Boston will be favorably considered, but the Buffalo school will endeavor to be represented at any time and place that the majority may deem expedient, provided *all* consent to the measure.

